# Influence of Drying Method on Some Bioactive Compounds and the Composition of Volatile Components in Dried Pink Rock Rose (*Cistus creticus* L.)

**DOI:** 10.3390/molecules25112596

**Published:** 2020-06-03

**Authors:** Natalia Matłok, Sabina Lachowicz, Józef Gorzelany, Maciej Balawejder

**Affiliations:** 1 Department of Food and Agriculture Production Engineering, University of Rzeszow, 4 Zelwerowicza St., 35-601 Rzeszów, Poland; gorzelan@ur.edu.pl; 2 Department of Technology Fermentation and Cereals, Wroclaw University of Environmental and Life Science, 37 Chelmonskiego Street, 51-630 Wroclaw, Poland; sabina.lachowicz@upwr.edu.pl; 3 Department of Chemistry and Food Toxicology, Collegium of Natural Sciences, University of Rzeszow, 1a Ćwiklińskiej St., 35-601 Rzeszów, Poland; maciejb@univ.rzeszow.pl

**Keywords:** *Cistus creticus* L., drying, flavonols, flavan-3-ols, hydrolyzed tannins, eugenol, thymol, carvacrol

## Abstract

This study investigates the effects of various drying methods applied to leaves of *Cistus creticus* L. on the contents of polyphenols and the composition of the volatile fraction. The following four drying methods were used: convection drying at a temperature of 40 °C (CD 40 °C), 50 °C (CD 50 °C), and 60 °C (CD 60 °C); vacuum-microwave (VMD 240 W); combined drying, involving convection pre-drying (50 °C) and vacuum-microwave (240 W) finish drying (CPD-VMFD) as well as freeze-drying (FD). Polyphenols in the dried leaves were determined using chromatography-photodiode detector-quadrupole/time of flight-mass spectrometry (UPLC-PDA-Q/TOF-MS). The contents of odoriferous substances in the dry material were determined by means of head space-solid phase microextraction (HS-SPME) with the use of a gas chromatograph (GC). Thirty-seven polyphenol components including 21 flavonols, eight flavan-3-ols, and eight hydrolyzed tannins in dry Pink Rock Rose material were found for the first time. The highest contents of polyphenols, totaling 2.8 g 100 g^−1^ dry matter (d.m.), were found in the samples subjected to the CPD/VMFD drying method. Pink Rock Rose subjected to this drying method was characterized by large quantities of odoriferous compounds, mainly eugenol, thymol, and carvacrol, which contribute to its antiseptic properties. By using CPD/VMFD methods, it is possible to obtain fine quality dry material from the leaves of *C. creticus*.

## 1. Introduction

The *Cistus* (family *Cistaceae*), also referred to as the rockrose, is a type of shrub growing in the Mediterranean region [1]. The genus *Cistus* comprises a number of species including *Cistus creticus*, *Cistus villosus*, *Cistus monspeliensis*, and *Cistus salviifolius* [2]. The plants classified in this genus are small coastal shrubs with pink or white flowers. They grow in very hot, sunny areas with calcareous soil as one of the degradation stages of evergreen forest plants [3].

*Cistus* plants are used in traditional folk medicine as anti-inflammatory, antiallergic, wound healing, antimicrobial, cytotoxic, and vasodilating agents. The beneficial properties of *Cistus* plants are mainly associated with the polyphenol content, compounds which are classified as strong antioxidants and radical scavengers [1]. Polyphenols inhibit the formation and development of inflammatory conditions in the body. Free radicals may contribute to the development of serious diseases such as cancer [4]. *Cistus infusions* are rich in phenolic acids and flavonoids (rosmarinic acid, quercetin, catechins, gallic acid) as a result of which they produce strong antioxidant effects [5]. During the Covid-19 epidemic, new antiviral drugs are being sought. Such action was found for a diethyl ether extract of oleoresin labdanum of *Cistus creticus* L. [6]. This activity was found in the treatment of a virulent hemorrhagic fever like dengue. Epi-manoyloxide, carvacrol, and labdanum based phytomedicine, isolated from *Cistus creticus* L., also shows high biological activity [7].

All the species of *Cistus* contain essential oils, but their composition depends on the cultivar conditions, type of soil, and time of harvest [8]. The contents of essential oils in *Cistus*, depending on the species, on average range from 0.09% in *Cistus creticus* to 0.12% in *Cistus villosus*, as calculated in dry matter [8]. Research findings also suggest there are significant differences in the composition of essential oils found in the same species of *Cistus*, and these seem to be linked to the habitat [9]. Although essential oils occur in all species of *Cistus*, it has also been reported that they cannot be extracted from some varieties [10].

The contents of bioactive compounds in the dry herbal product of *Cistus creticus* depend on the method of thermal processing applied. A study by Stępień et al. [11] showed that the processing method and drying temperature applied affect the total contents of polyphenols in, and the antioxidative potential of, the final product.

The purpose of the study was to determine the effects of the drying method and conditions on the content and composition of polyphenols and the composition of the volatile headspace fraction of Pink Rock Rose (*Cistus creticus* L.) by using convection drying (CD) at 40 °C (CD 40 °C), 50 °C (CD 50 °C), and 60 °C (CD 60 °C); vacuum-microwave (VMD 240 W); and combined drying, involving convection pre-drying (50 °C), vacuum-microwave (240 W) finish drying (CPD/VMFD), and freeze-drying (FD).

## 2. Results and Discussion

### 2.1. Polyphenolic Compounds

The UPLC-PDA-Q/TPF-MS technique permitted the identification of 37 polyphenol components including flavonols (21 components), flavan-3-ols (eight components), and hydrolyzed tannins (eight components) (Table 1). The polyphenolic compound group was evaluated in the order: flavonols (31–48%) > flavan-3-ols (37–42%) ≫ hydrolyzed tannins (7–18%). The amounts of polyphenols recorded in dried *Cistus creticus* samples after the FD to CPD/VMFD drying method were from 1.9 g 100 g^−1^ d.m. to 2.8 g 100 g^−1^ d.m. The concentration of polyphenols differed significantly, depending on the drying method. Loizzo et al. [8] investigated Pink Rock Rose from natural habitats. In nature, the growing conditions like the soil, weather, and influence of other plants determine the polyphenol content. In order to eliminate these variables, tests should be carried out on cultivated plants. It should be noted that the application of the combined method may influence the final chemical composition of the dried herbal product. The use of the CPD-VMFD combination drying method produces the highest quality dried basil [12] and thyme [13]. The lowest loss of polyphenols was noted after combined drying (CPD/VMFD). These differences could be observed due to the degradation of the polyphenols or the ability to extract them from plant tissues when dried by different methods [14]. No extra metabolites were found in the extracts, so probably the differences in polyphenol content are observed due to extraction susceptibility. For this drying method, a similar conclusion was noted for Rock Rose leaves [11], which produced the highest concentration of these components. The greatest degradation of polyphenols was found after FD, which produced around thirty percent less that the CPD/VMFD method. The test samples after CD 40 °C contained the highest total retention of polyphenols, which was between 1.5% and 2.5% higher than convection drying at 50 °C and 60 °C, respectively. Similar observations were found after the drying of Rock Rose leaves [11], or blueberry [15]. It is possible that irreversible hot air, a long process time, and oxidation negatively affect the degradation of bioactive compounds [11,16] in *C. creticus*.

Flavanols were identified as the most abundant group of polyphenolic compounds in *C. creticus.* The content of these compounds ranged between 0.8 and 1.3 g 100 g^−1^ d.m. for a sample subject to the CD 60 °C drying method after FD. A total of 21 flavonols were detected including myricetin (average 85.9% of all flavonols) ≫ quercetin (4.8%) > kaempferol (1.6%) ≫ lutein (0.5%) > isorhamnetin (0.2%) derivatives ([M − H]^−^ at *m*/*z* 317, 301, 285, 315, respectively). We noted the most common derivatives for myricetin as 3-*O*-galactoside, 3-*O*-glucoside (*m*/*z* 479), 3-*O*-rhamnoside (*m*/*z* 463), and 3-*O*-rutinoside (*m*/*z* 625 and 479); for quercetin as 3-*O*-galactoside, 3-*O*-glucoside (*m*/*z* 463), and 3-*O*-rhamnoside (*m*/*z* 447), 3-*O*-(6′′-acetyl)arabinopyranoside (*m*/*z* 475); for kaempferol as 3-*O*-diglucoside (*m*/*z* 609) and 3-*O*-rutinoside (*m*/*z* 593), and for lutein and isorhamnetin as 3-*O*-rutinoside (*m*/*z* 593 and 475) and 3-*O*-glucoside (*m*/*z* 477). These compounds were previously described by other authors [17,18,19] in chokeberry, saskatoon berry, and apricot, but were observed in *C. creticus* for the first time. Compound nos. 18, 29, and 34 demonstrated MS/MS fragmentation to flavonols. This testifies to the fact that they could be quercetin derivatives (*m*/*z* 301). These compounds were detected as quercetin-di-hexoside, quercetin-rhamnoside-dihexoside, and quercetin-deoxyhexoside-hexoside. The first peak gives a [M − H]^−^ ion at *m*/*z* 625 and MS/MS fragment at *m*/*z* 463 ([M − H − 162 − 162]^−^), and the loss of two −162 Da as hexose units. The second peak had a main ion at *m*/*z* 771, and fragmentation at *m*/*z* 625, and the loss of the rhamnose (−146 Da) and two times hexose (−162 Da) moiety. There is a third peak of [M − H]^−^ at *m*/*z* 755, with a corresponding loss of rhamnose (−146 Da) and deoxyhexose (−308 Da). Compound nos. 17 and 30 point to myricetin derivatives (*m*/*z* 317), and had a major peak at *m*/*z* 771, and MS/MS fragmentation yielded [M − H − 162 − 146 − 146]^−^ and [M − H − 146 − 308]^−^, which probably indicates a loss of hexose and two moieties, a rhamnose moiety and a rhamnose and deoxyhexose moiety. These compounds were tentatively characterized as myricetin-hexoside-di-rhamnoside and myricetin deoxyhexoside-hexoside. Compound nos. 21 and 22 were based on the major ion at *m*/*z* 739 and 901, and were tentatively noted as luteolin-hexoside-dirhamnoside, and luteolin-dihexoside-dirhamnoside. These peaks fragment at *m*/*z* 577 and 285, and *m*/*z* 739 and 285, showing a loss of −162 Da and two −146 Da units as hexose and two rhamnose moieties, and two −162 Da and two −146 Da units as two hexose and two rhamnose moieties. These seven compounds were noted in *C. creticus* for the first time.

The predominant compounds in *C. creticus* were myricetin 3-*O*-rhamnoside, which accounted for 80–85% of all flavonols. The average content was 9.4 g 100 g^−1^ d.m., while the highest amount of this compound was noted in the sample after CD 60 °C and the lowest after the FD drying method. The combined drying method (CPD/VMFD) was comparable to the CD 60 °C. However, it was observed in the application of the convection method that the higher the temperature, the higher the content of the compounds tested. This suggests that these methods may affect the higher retention of these compounds [20].

The group of ten polyphenols next measured in *C. creticus* was that of the flana-3-ols, and eight of their derivatives such as galloyl, prodelphinidin, (+)catechin, and (−)epicatechin (*m*/*z* 331, 305, and 289, respectively) were detected. Their content ranged from 1.0 (FD drying method) to 1.2 g 100 g^−1^ d.m. (CPD/VMFD). All compounds detected in this group were previously confirmed by other authors in pomegranate husk, saskatoon berry, cranberry, and *Myrciaria jaboticaba* peel [21,22,23,24]. The concentration of the compounds analyzed showed a significant dependence on the drying method used. The main fraction of these groups was made up of polymeric procyanidins (47–60% of all flavan-3-ols identified). Higher retention of these compounds was found after FD, with the lowest after VMD. On the other hand, the lowest degradation of flavan-3-ols (monomers and oligomers) was noted after VMD, and the lowest retention of these compounds was found in the sample after FD. Similar observations were found after the drying of plum [20], and the contrary with saskatoon berry [22]. It could be that high temperature, a long process time, and/or oxidation have an impact on the low retention and high degradation of these compounds [16] in *C. creticus*.

The last and smallest polyphenolic group determined in *C. creticus* was that of hydrolyzed tannins (*m*/*z* 301) (Table 1). All the compounds detected in this group were previously confirmed by other authors in *Myrciaria jaboticaba* and *Duchesnea indica* [23,24,25,26], but were found in *C. creticus* for the first time. The average amount of hydrolyzed tannins was 0.4 g 100 g^−1^ d.m., and the highest retention of these compounds was noted in the sample after (CPD/VMFD), but degradation was observed after the FD method (around 60% compared to CPD/VMFD). The content of the test compounds showed a significant dependence on the drying methods used. A greater degradation of hydrolyzed tannins was noted after VMD and CD 60 °C, around 17 and 20%, respectively. The major components of hydrolyzed tannins were *bis*-HHDP-glucose (*m*/*z* 783), and cornusiin B (*m*/*z* 1085), which accounted for 55% and 10% of all hydrolyzed tannins.

### 2.2. Chemical Composition of HS-SPME

**Figure 1:**
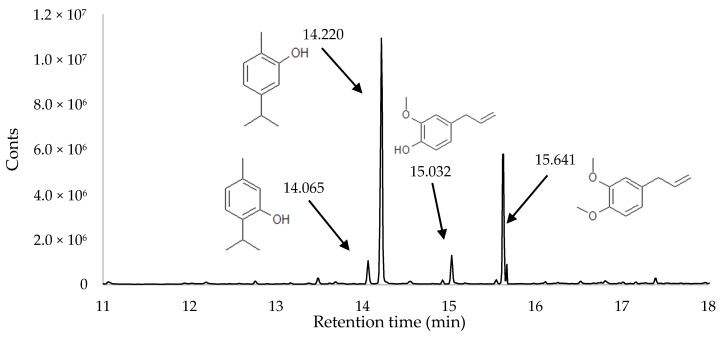
SPME-GC chromatograms for the volatile fraction of dry material from Pink Rock Rose (*Cistus creticus* L.) (CPD/VMFD).

The tests focusing on the volatile components started with an attempt to isolate essential oils using a hydrodistillation technique [27]. Despite a number of attempts, no essential oil was isolated from 200 g of the dry *Cistus* material. According to the relevant literature, the contents of essential oil in *Cistus* leaves vary depending on the cultivar, type of soil, and time of harvest. Loizzo et al. [8] reported a total content of essential oil in *C. creticus* of 0.09%. Additionally, it was shown that essential oils cannot be extracted from some varieties of *Cistus* [8]. In the case of the dry material obtained, HS-SPME seems to be the only method by which it would be possible to examine volatile compounds. The main components of the volatile fraction in the headspace were determined to be the following: thymol, carvacrol, and eugenol as well as methyl eugenol. Some of the compounds identified also occur in essential oils (e.g., linalool, decanal, eugenol, and methyl eugenol) [28]. The remaining components such as thymol and carvacrol have a high boiling point and were not removed from the material during the drying process (Figure 1, Table 2). The main components of *Cistus* essential oil that were identified were 13-epi-manoyl oxide and manool [28], which cannot always be determined using HS-SPME [29]. Due to their high boiling point (over 350 °C), and consequent poor volatility, these compounds do not significantly affect the aroma of the raw material.

## 3. Materials and Methods

### 3.1. Plant Materials

The material used in the study consisted of dry leaves of Pink Rock Rose (*Cistus creticus* L.) (specimen voucher CONN00052561). Leaves of *C. creticus* were processed using the following methods: convection drying (CD) at 40 °C (CD 40 °C), 50 °C (CD 50 °C), and 60 °C (CD 60 °C); vacuum-microwave (VMD 240 W); combined drying, involving convection pre-drying (50 °C), vacuum-microwave (240 W) finish drying (CPD/VMFD), and freeze-drying (FD) [11]. Convection drying was performed in a dryer installed in the Institute of Agricultural Engineering (WUELS, Wroclaw, Poland). The air stream velocity was 0.8 m s^−1^. The VMD process was performed in a Plazmatronika SM 200 dryer (UniClever II, Wroclaw, Poland) at the constant magnetron output power of 240 W. The samples were placed in a 6.8-L Plexiglas container. The pressure was reduced during the drying process to 4 to 6 kPa. The CPD-VMFD method involved pre-drying of the samples for 3 h in a convection dryer at a temperature and airflow of 50 °C and 0.8 m s^−1^, respectively, and subsequently, additional drying was performed in a vacuum-microwave dryer at a power of 240 W magnetrons. The procedure was performed in three replications. The mean content of water in the dry material from *C. creticus* was 7.2% (CD 40 °C = 7.8%; CD 50 °C = 7.3; CD 60 °C = 7.5%; VMD = 0.4%; CPD/VMFD = 7.4%). After drying, the plant materials were ground and stored in a fridge (DANLAB, Białystok, Poland) at 4 °C and hermetic plastic bags were utilized for further analyses.

### 3.2. Identification and Quantification of Polyphenolic Compounds

The test of polyphenols present in *Cistus creticus* L. subjected to dehydration by FD, CD 40 °C, CD 50 °C, CD 60 °C, VMD, and CPD/VMFD were carried out using an ACQUITY Ultra Performance Liquid Chromatography system (Waters Corporation, Milford, MA, USA) with a binary solvent manager and PDA detector coupled to G2 Q/TOF micro-mass spectrometer (Waters, Manchester, UK) fitted with an electrospray ionization (ESI) source acting on negative and positive modes. The method was performed in the manner described by Lachowicz et al. [22]. The runs were monitored at the following wavelengths: flavonols at 360 nm, hydrolyzed tannins 240 nm, flavan-3-ols at 280 nm. Separations of individual polyphenols were carried out using an UPLC BEH C18 column (1.7 μm, 2.1 × 100 mm, Waters Corporation, Milford, MA) at 30 °C. The samples (10 μL) were injected, and the elution was completed in 15 min with a sequence of linear gradients and isocratic flow rates of 0.45 mL/min. The mobile phase consisted of solvent A (2.0% formic acid, *v*/*v*) and solvent B (100% acetonitrile). The program began with isocratic elution with 99% solvent A (0–1 min), and then a linear gradient was used until 12 min, lowering solvent A to 0%; from 12.5 to 13.5 min, the gradient returned to the initial composition (99% A), and then it was held constant to re-equilibrate the column. The optimized MS parameters were as follows: source temperature of 100 °C, desolvation temperature of 300 °C, cone gas flow 40 L/h, desolvation gas flow 300 L/h, capillary voltage of 2500 V, and cone voltage of 30 V. The MS analysis was performed using mass scanning from *m*/*z* 100 to 1200. The PDA spectra were measured over the wavelength range of 200–600 nm in steps of 2 nm. All data were obtained in triplicate. The calibration curves were run at 360 nm for the standard keampferol-3-*O*-galactoside, quercetin-3-*O*-galactoside, luteolin-3-*O*-rutinoside; at 240 nm for the standard ellagic acid; and at 280 nm for the standards (−)-epicatechin, (+)-catechin, procyanidins B2, at concentrations ranging from 0.05–5 mg mL^−1^ (R^2^ = 0.9999). SD. The results of the identification process are presented in Table 1. The results were expressed as mg 100 g^−1^ (also g 100 g^−1^) dry matter (d.m.). The analyses were in triplicate and results are given as a mean value ± SD.

### 3.3. Analysis of Proanthocyanidins by Phloroglucinolysis

Direct phloroglucinolysis of samples in *Cistus creticus* L. subjected to dehydration by FD, CD 40 °C, CD 50 °C, CD 60 °C, VMD, and CPD/VMFD was performed as described by Lachowicz et al. [22] by the HPLC system. Phloroglucinolysis products were separated on a Cadenza CD C18 (75 mm × 4.6 mm, 3 μm) column (Imtakt, Kyoto, Japan). The liquid chromatograph was a Waters (Milford, MA, USA) system equipped with a diode array and scanning fluorescence detectors (Waters 474) and autosampler (Waters 717 plus). Solvent A (25 mL acetic acid and 975 mL water) and solvent B (acetonitrile) were used in the following gradients: initial, 5 mL/100 mL B; 0–15 min, to 10 mL/100 mL B linear; 15–25 min to 60 mL/100 mL B linear; followed by washing and reconditioning of the column. The flow rate was 1 mL/min and the oven temperature was 15 °C with the injection of the filtrate (20 μL) on the HPLC system. The fluorescence detection was carried out at an excitation wavelength of 278 nm and emission wavelength of 360 nm. The calibration curves, which were based on peak area, were established using (+)-catechin, (−)-epicatechin, (+)-catechins and (−)-epicatechin-phloroglucinol adduct standards. The average degree of polymerization was measured by calculating the molar ratio of all the flavan-3-ol units (phloroglucinol adducts + terminal units) to (−)-epicatechin and (+)-catechin, which correspond to terminal units. Quantification of the (+)-catechin, (−)-epicatechin, (+)-catechin, and (−)-epicatechin-phloroglucinol adducts was achieved by using the calibration curves of the corresponding standards (Extrasynthese). The analyses were in triplicate and the results are given as a mean value ± SD. The results are shown in Table 1 and expressed as mg 100 g^−1^ d.m.

### 3.4. Head Space-Solid Phase Microextraction (HS-SPME)

The dry material was subjected to head space-solid phase microextraction (HS-SPME) analysis, with the use of 100 μm polydimethylsiloxane (PDMS) fiber manufactured by Supelco Ltd. (Bellefonte, PA, USA). Prior to the analyses, the fiber was conditioned in the injector of a gas chromatograph (Varian Instrument Group, Sunnyvale, CA, Canada) at 250 °C for 30 min in accordance with the manufacturer’s instructions. The material being analyzed, with a weight of 3 ± 0.01 g, was placed in a 100 mL glass vial, and sealed with a cap provided with silicone septa. Exposure of the fiber in the headspace phase of the samples took place for 30 s at a temperature of 20 °C. Subsequently, the fiber was transferred to the injector of the gas chromatograph (temp. 250 °C), where the analytes were thermally desorbed for a duration of 5 min. The composition of the compounds desorbed from the SPME fiber was examined using a gas chromatograph (GC-MS, Varian 450GC coupled with 240 MS) [27]. The analyses were in triplicate and the results given as a mean value ± SD.

### 3.5. Chromatographic Analysis HS-SPME

The composition of the desorbed compounds was examined using a Varian 450 GC gas chromatograph with the Varian 240 MS mass detector (Varian Instrument Group, Sunnyvale, CA, Canada). The carrier gas applied was helium, with a flow rate of 1 mL min^−1^ and injector temperature of 250 °C. Separation of analytes was conducted using a 30 m × 0.25 mm capillary column with a moderately polar stationary phase HP-5 (methyl phenyl polysiloxane), with a film thickness of 0.25 μm. The column oven temperature program started at 50 °C (5 min of isotherm), followed by an increase in temperature at the rate of 10 °C min^−1^ up to 300 °C (5 min of isotherm). The analysis was continued for 35 min. The compounds were identified based on the NIST.08 and Willey database.

### 3.6. Statistical Analysis

The statistical analysis of the results obtained was conducted using the Statistica program, version 13.1 (TIBCO Software Inc., Hillview Avenue, Palo Alto, CA, USA). The significances of differences between results were determined by analysis of variance at a significance level α = 0.05.

## 4. Conclusions

Dry material from Pink Rock-Rose (*Cistus creticus* L.) is a good source of bioactive compounds; the substances identified included 37 compounds from the polyphenol group such as flavonols (21 components), flavan-3-ols (eight components), and hydrolyzed tannins (eight components). The highest contents of polyphenols, totaling 2.8 g 100 g^−1^ d.m., were found in the samples subjected to the CPD/VMFD drying method. However, despite the lower contents of bioactive compounds in the other samples of dry *Cistus* material, compared to the dry material obtained using the CPD/VMFD method, all forms may potentially be used in functional food or pharmaceuticals. Although it was subjected to a drying process, the material retained volatile compounds, which contributed to the sensory attractiveness of the final herbal product. In addition, the eugenol, thymol, and carvacrol contents in the acquired dry material favorably affect its antiseptic properties.

## Figures and Tables

**Table 1:** Polyphenolic content of Pink Rock Rose (*Cistus creticus* L.)**.**

Peak No		Tentative Compounds	[M − H]^−^ (*m*/*z*)/[M − H]^−^ MS/MS (*m*/*z*)	Δ_max_ [nm]	Rt [min]	VMD	CPD/VMFD	CD 40 °C	CD 50 °C	CD 60 °C	FD
**Flavonols**	17	Myricetin-hexoside-di-rhamnoside	771/609/317	355	6.03	4.5 ± 0.1 ^b^	5.8 ± 0.1 ^c^	6.0 ± 0.1 ^d^	5.5 ± 0.1 ^c^	5.6 ± 0.1 ^c^	3.2 ± 0.1 ^a^
	18	Quercetin-di-hexoside	625/463/301	356	6.59	19.0 ± 0.4 ^b^	19.7 ± 0.4 ^b^	19.1 ± 0.4 ^b^	19.7 ± 0.4 ^b^	19.4 ± 0.4 ^b^	17.3 ± 0.3 ^a^
	19	Myricetin-3-*O*-galactoside	479/317	358	6.69	9.1 ± 0.2 ^a^	10.5 ± 0.2 ^b^	12.5 ± 0.2 ^c^	12.2 ± 0.2 ^c^	12.6 ± 0.3 ^c^	10.1 ± 0.2 ^b^
	20	Myricetin-3-*O*-glucoside	479/317	354	6.78	12.8 ± 0.3 ^b^	12.9 ± 0.3 ^b^	12.3 ± 0.2 ^b^	12.1 ± 0.2 ^b^	15.1 ± 0.3	8.8 ± 0.2 ^a^
	21	Luteolin-hexoside-di rhamnoside	739/577/285	350	7.06	1.1 ± 0.01 ^a^	1.1 ± 0.01 ^a^	1.1 ± 0.01 ^a^	1.0 ± 0.01 ^a^	1.1 ± 0.01 ^a^	1.2 ± 0.01 ^a^
	22	Luteolin-dihexoside-dirhamnoside	901/739/285	350	7.30	3.5 ± 0.1 ^a^	3.6 ± 0.1 ^a^	3.6 ± 0.1 ^a^	3.5 ± 0.1 ^a^	4.0 ± 0.1 ^b^	3.3 ± 0.1 ^a^
	23	Quercetin-3-*O*-rutinoside	609/301	353	7.37	55.9 ± 1.1 ^b^	58.4 ± 1.2 ^b^	55.6 ± 1.1 ^b^	58.4 ± 1.2 ^b^	57.2 ± 1.1 ^b^	44.6 ± 0.9 ^a^
	24	Myricetin-3-*O*-rhamnoside	463/317	350	7.44	969.6 ± 19.4 ^b^	1032.2 ± 20.6 ^c^	964.4 ± 19.3 ^b^	968.8 ± 19.4 ^b^	1046 ± 20.9 ^c^	631.5 ± 12.6 ^a^
	25	Quercetin-3-*O*-galactoside	463	350	7.55	2.1 ± 0.01 ^a^	2.9 ± 0.1 ^b^	3.0 ± 0.1 ^b^	3.4 ± 0.1 ^c^	2.9 ± 0.1 ^b^	3.8 ± 0.1 ^c^
	26	Quercetin-3-*O*-glucoside	463	350	7.70	1.2 ± 0.01 ^a^	1.7 ± 0.01 ^b^	1.2 ± 0.01 ^a^	1.7 ± 0.01 ^b^	1.8 ± 0.01 ^b^	1.4 ± 0.01 ^a^
	27	Luteolin-3-*O*-rutinoside	593/475/285	349	8.09	1.3 ± 0.01 ^a^	1.3 ± 0.01 ^a^	1.4 ± 0.01 ^a^	1.3 ± 0.01 ^a^	1.3 ± 0.01 ^a^	1.4 ± 0.01 ^a^
	28	Quercetin-3-*O*-(600-acetyl)arabinopyranoside	475/301	351	8.19	2.3 ± 0.01 ^a^	2.5 ± 0.01 ^a^	3.2 ± 0.1 ^b^	2.6 ± 0.1 ^a^	2.7 ± 0.1 ^a^	3.1 ± 0.1 ^b^
	29	Quercetin-rhamnoside-dihexoside	771/625/301	351	8.33	2.3 ± 0.01 ^a^	2.7 ± 0.1 ^b^	2.7 ± 0.1 ^b^	3.1 ± 0.1 ^c^	3.1 ± 0.1 ^c^	2.4 ± 0.01 ^a^
	30	Myricetin deohyhexoside-hexoside	771/625/317	351	8.43	18.2 ± 0.4 ^a^	20.8 ± 0.4 ^b^	20.1 ± 0.4 ^b^	21.2 ± 0.4 ^b^	24.2 ± 0.5 ^c^	18.8 ± 0.4 ^a^
	31	Quercetin-3-*O*-rhamnoside	447/301	350	8.51	23.9 ± 0.5 ^b^	26.0 ± 0.5 ^c^	24.9 ± 0.5 ^b^	25.8 ± 0.5 ^c^	28.4 ± 0.6 ^d^	20.5 ± 0.4 ^a^
	32	Isorhamnetin-3-*O*-glucoside	477/315	359	8.62	2.3 ± 0.01 ^a^	2.3 ± 0.01 ^a^	2.0 ± 0.01 ^a^	3.2 ± 0.1 ^c^	3.6 ± 0.1 ^c^	2.5 ± 0.1 ^b^
	33	Kaempferl-3-*O*-diglucoside	609/285	370	9.04	2.0 ± 0.01 ^b^	2.2 ± 0.01 ^b^	1.8 ± 0.01 ^b^	2.0 ± 0.01 ^b^	2.9 ± 0.1 ^c^	1.3 ± 0.01 ^a^
	34	Quercetin-deoxyhexoside-hexoside	755/609/301	369	9.10	1.1 ± 0.01 ^a^	1.2 ± 0.01 ^a^	1.1 ± 0.01 ^a^	1.1 ± 0.01 ^a^	1.2 ± 0.01 ^a^	1.0 ± 0.01 ^a^
	35	Myricetin-3-*O*-rutinoside	625/479/317	363	9.51	2.3 ± 0.01 ^a^	2.4 ± 0.01 ^a^	2.5 ± 0.1 ^a^	2.6 ± 0.1 ^b^	2.9 ± 0.1 ^c^	2.3 ± 0.01 ^a^
	36	Kaempferl-3-*O*-rutinoside	593/285	354	10.95	12.0 ± 0.2 ^a^	14.1 ± 0.3 ^c^	13.4 ± 0.3 ^b^	14.3 ± 0.3 ^c^	14.1 ± 0.3 ^c^	12.8 ± 0.3 ^a^
	37	Kaempferl-3-*O*-rutinoside	593/285	354	11.20	2.7 ± 0.1 ^c^	2.4 ± 0.01 ^b^	2.5 ± 0.1 ^b^	2.5 ± 0.01 ^b^	2.8 ± 0.1 ^c^	2.2 ± 0.01 ^a^
Sum of flavonols (mg 100 g^−1^)						1149.3 ^b^	1226.7 ^b^	1154.6 ^b^	1165.8 ^b^	1252.9 ^b^	793.6 ^a^
Sum of flavonols (g 100 g^−1^)						1.2 ^b^	1.2 ^b^	1.2 ^b^	1.2 ^b^	1.3 ^b^	0.8 ^a^
**Flavan-3-ols**	1	Gaolloyl glucose	331	266	2.08	156.98 ± 3.14 ^b^	110.85 ± 2.22 ^a^	95.83 ± 1.92 ^a^	113.93 ± 2.28 ^a^	108.82 ± 2.18 ^a^	100.7 ± 2.01 ^a^
	2	Gallocatechintrimer	913/609/423/305	280	2.74	22.35 ± 0.45 ^c^	23.39 ± 0.47 ^c^	24.1 ± 0.48 ^c^	20.98 ± 0.42 ^b^	20.24 ± 0.4 ^b^	10.51 ± 0.21 ^a^
	3	Gallocatechin dimer	609/423/305	270	2.83	18.42 ± 0.37 ^b^	20.17 ± 0.4 ^c^	20.73 ± 0.41 ^c^	17.42 ± 0.35 ^b^	17.97 ± 0.36 ^b^	6.78 ± 0.14 ^a^
	4	(+)-catechin	289	270	3.14	31.68 ± 0.63 ^b^	36.95 ± 0.74 ^c^	36.52 ± 0.73 ^c^	31.57 ± 0.63 ^b^	32.92 ± 0.66 ^b^	26.02 ± 0.52 ^a^
	6	Gallocatechin	305	270	3.31	172.89 ± 3.46 ^c^	188.3 ± 3.77 ^c^	166.84 ± 3.34 ^b^	169.61 ± 3.39 ^b^	156.73 ± 3.13 ^b^	72.16 ± 1.44 ^a^
	7	Prodelphinidin dimer	593/305	286	3.57	44.79 ± 0.9 ^a^	48.48 ± 0.97 ^b^	49.31 ± 0.99 ^b^	48.18 ± 0.96 ^b^	49.37 ± 0.99 ^b^	76.48 ± 1.53 ^c^
	13	(−)-epicatechin	289	277	4.78	92.29 ± 1.85 ^b^	118.81 ± 2.38 ^c^	115.73 ± 2.31 ^c^	111.45 ± 2.23 ^c^	102.35 ± 2.05 ^b^	73.88 ± 1.48 ^a^
	16	Gallocatechin-(4α-8)-catechin	593/289	291	5.78	12.61 ± 0.25 ^a^	13.71 ± 0.27 ^b^	22.34 ± 0.45 ^c^	13.01 ± 0.26 ^b^	10.48 ± 0.21 ^a^	16.39 ± 0.33 ^b^
		Polymers proanthocyanidins				497.56 ± 9.95 ^a^	511.31 ± 10.23 ^a^	563.95 ± 11.28 ^b^	553.74 ± 11.07 ^b^	508.4 ± 10.17 ^a^	570.98 ± 11.42 ^b^
Sum of flavan-3-ols (mg 100 g^−1^)						1049.8 ^b^	1072.0 ^b^	1095.4 ^b^	1080.0 ^b^	1007.3 ^a^	953.9 ^a^
Sum of flavan-3-ols (g 100 g^−1^)						1.1 ^b^	1.1 ^b^	1.1 ^b^	1.1 ^b^	1.0 ^a^	1.0
**Hydrolyzed tannins**	5	*bis*-HHDP-glucose	783/481/301	218	3.25	237.0 ± 4.7 ^b^	263.7 ± 5.3 ^c^	256 ± 5.1 ^c^	231.6 ± 4.6 ^b^	221.9 ± 4.4 ^b^	89.4 ± 1.8 ^a^
	8	Ellagic acid rhamnoside	633/593/301	208	3.58	20.1 ± 0.4 ^b^	20.7 ± 0.4 ^b^	24.4 ± 0.5 ^c^	19.4 ± 0.4 ^a^	19.1 ± 0.4 ^a^	18.8 ± 0.4 ^a^
	9	Ellagitannin	933/783/609/423/305/301	208	3.79	15.8 ± 0.3 ^b^	16.7 ± 0.3 ^b^	16.2 ± 0.3 ^b^	14.3 ± 0.3 ^b^	16.6 ± 0.3 ^b^	5.7 ± 0.1 ^a^
	10	*bis*-HHDP-glucose	783/481/301	226	4.15	26.9 ± 0.5 ^b^	33.7 ± 0.7 ^c^	28.2 ± 0.6 ^b^	29.7 ± 0.6 ^c^	24.3 ± 0.5 ^b^	16.4 ± 0.3 ^a^
	11	Punicalagin isomer	1083/781/601/301	250	4.63	10.0 ± 0.2 ^a^	19.6 ± 0.4 ^b^	20.4 ± 0.4 ^b^	17.6 ± 0.4 ^b^	12.3 ± 0.2 ^a^	9.3 ± 0.2 ^a^
	12	Cornusiin B	1085/783/451/301	240	4.75	24.3 ± 0.5 ^b^	27.2 ± 0.5 ^b^	27.6 ± 0.6 ^b^	26.5 ± 0.5 ^b^	27.5 ± 0.5 ^b^	17.7 ± 0.4 ^a^
	14	Cornusiin B	1085/783/451/301	240	4.87	42.8 ± 0.9 ^b^	67.8 ± 1.4 ^c^	67.4 ± 1.3 ^c^	68.5 ± 1.4 ^c^	46.5 ± 0.9 ^b^	30.5 ± 0.6 ^a^
	15	Ellagic acid rutinoside	609/463/301	241	5.49	18.9 ± 0.4 ^b^	23.6 ± 0.5 ^c^	25.3 ± 0.5 ^c^	21.4 ± 0.4 ^b^	20.6 ± 0.4 ^b^	3.7 ± 0.1 ^a^
Sum of hydrolyzed tannins (mg 100 g^−1^)						395.8 ^b^	473.1 ^c^	465.5 ^c^	429.0 ^b^	388.8 ^b^	191.5 ^a^
Sum of hydrolyzed tannins (g 100 g^−1^)						0.4 ^b^	0.5 ^c^	0.5 ^c^	0.4 ^b^	0.4 ^b^	0.2 ^a^
Sum of phenolic compounds (mg 100 g^−1^)						2594.7 ^b^	2771.7 ^b^	2715.4 ^b^	2674.8 ^b^	2649.0 ^b^	1938.0 ^a^
Sum of phenolic compounds (g 100 g^−1^)						2.6 ^b^	2.8 ^b^	2.7 ^b^	2.7 ^b^	2.7 ^b^	1.4 ^a^

RT—retention time; ± SD and *n* = 3; FD, freeze-drying; VMD, vacuum-microwave drying at 240 W; (CPD/VMFD)—combined drying (of convection pre-drying and convection-microwave drying at 50 °C and vacuum at 240 W); CD 40 °C, CD 50 °C, CD 60 °C, convection drying (at 40, 50, 60 °C). Note. a, b, c are the mean values within rows where different letters differ significantly at *p* ≤ 0.05.

**Table 2:** Chemical composition of the headspace fraction HS-SPME in dry material from Pink Rock Rose (*Cistus creticus* L.)**.**

No.	Ordinary Substance Name	Systematic Substance Name	RT [min]	Peak Share in the Chromatogram [%]						No CAS
				VMD	CPD/VMFD	CD 40 °C	CD 50 °C	CD 60 °C	FD	
1	linalool	(±)-3,7-Dimethyl-1,6-octadien-3-ol, (±)-3,7-Dimethyl-3-hydroxy-1,6-octadiene	10.99	trace	trace	trace	trace	trace	trace	78-70-6
2	decanal	capraldehyde	12.79	trace	trace	trace	trace	trace	trace	112-31-2
3	thymoquinone	2-isopropyl-5-methyl-1,4-benzoquinone	13.48	1.65 ± 0.3 ^a^	1.94 ± 0.4 ^c^	1.75 ± 0.2 ^b^	1.80 ± 0.3 ^b^	1.49 ± 0.1 ^a^	1.64 ± 0.2 ^a^	490-91-5
4	thymol	2-isopropyl-5-methylphenol	14.06	5.37 ± 0.6 ^a^	5.04 ± 0.5 ^a^	5.50 ± 0.6 ^a^	6.05 ± 0.3 ^b^	5.51 ± 0.7 ^a^	5.51 ± 0.2 ^a^	89-83-8
5	carvacrol	5-isopropyl-2-methylphenol	14.22	58.8 ± 1.1 ^a^	61.9 ± 1.3 ^c^	58.47 ± 1.7 ^a^	62.66 ± 1.6 ^c^	60.26 ± 1.7 ^b^	59.48 ± 1.4 ^b^	499-75-2
6	alpha-terpinyl acetate	2-(4-methyl-3-cyclohexen-1-yl)-2-propanyl acetate	14.92	trace	trace	1.31 ± 0.1 ^a^	trace	trace	trace	80-26-2
7	eugenol	4-allyl-1-hydroxy-2-methoxybenzene	15.03	6.72 ± 0.5 ^b^	6.91 ± 0.3 ^b^	6.92 ± 0.1 ^b^	4.551 ± 0.3 ^a^	6.55 ± 0.7 ^b^	6.32 ± 0.4 ^b^	97-53-0
8	methyl eugenol	4-allyl veratrole	15.64	24.63 ± 0.8 ^a^	24.14 ± 0.9 ^a^	24.60 ± 1.3 ^a^	24.89 ± 0.7 ^a^	24.55 ± 0.6 ^a^	24.31 ± 0.3 ^a^	93-15-2
9	dihydroactinidiolide	(*S*)-dihydroactinidiolide	17.39	trace	trace	trace	trace	trace	trace	17092-92-1
TOTAL			97.21 ^a^	99.99 ^b^	98.55 ^a^	99.96 ^b^	98.36 ^a^	97.27 ^a^		

RT—retention time; ± SD and *n* = 3; FD, freeze-drying; VMD, vacuum-microwave drying at 240 W; (CPD/VMFD)—combined drying (of convection pre-drying and convection-microwave drying at 50 °C and vacuum at 240 W); CD 40 °C, CD 50 °C, CD 60 °C, convection drying (at 40, 50, 60 °C;) Note. a, b, c are the mean values within rows where different letters differ significantly at *p* ≤ 0.05.
